# The best way to skin a cat: product consumption versus direct observation for monitoring hand hygiene performance

**DOI:** 10.1186/1753-6561-5-S6-P103

**Published:** 2011-06-29

**Authors:** AJ Stewardson, H Attar, S Touveneau, W Zingg, S Longet-Di Pietro, N Vernaz, D Pittet, H Sax, Members of the Infection Control Program

**Affiliations:** 1 University of Geneva Hospitals, Geneva, Switzerland

## Introduction / objectives

Direct observation (DO) and alcohol-based handrub (ABHR)Â consumption per 1000 patient daysÂ (AC) are used to monitor hand hygiene (HH) performance in healthcare, and are increasingly utilised as quality indicators, sometimes for external benchmarking. We investigated the common assumption that there is a direct correlation between these two measures.

## Methods

ForÂ the baseline period of a cluster-randomized trial regarding multimodal hand hygiene promotion at a 2200 bed tertiary-care facility, 8 validated infection control nurses performed DO using the WHO ‘My 5 Moments’ method in 65 non-ICU acute care wards for 15 months from April 2009.Â ABHR usage and patient days per ward were extracted from hospital databases to calculate AC over the same period. Linear regression was used to determine the correlation between these two variables, with each unit’s compliance weighted for the number of HH opportunities observed.

## Results

DO capturedÂ 4601 HH opportunities and 2962 HH actions, 99% of which involved ABHR use. HH compliance in individual wards ranged fromÂ 38.2% toÂ 90.2% with a mean of 64.6%, (CI95, 62.0-67.2).Â A total of 13,939 litresÂ of ABHR was consumed during 459,917 patient days. AC ranged from 10.8 to 62.0 L/1000 bed days with a mean of 31.4 (CI95, 28.5-34.4). Both HH compliance and AC were normally distributed, with a weak and non-significant correlation (r=0.13; *P*=0.21).

## Conclusion

In this setting, DO and AC are not significantly correlated, complicating efforts to monitor HH performance. Further investigation should examine which is a better indicator for relevant clinical outcomes such as microbial colonisation and healthcare-associated infection.

## Disclosure of interest

None declared.

